# Near-infrared bioluminescent proteins for two-color multimodal imaging

**DOI:** 10.1038/srep36588

**Published:** 2016-11-11

**Authors:** Konstantin A. Rumyantsev, Konstantin K. Turoverov, Vladislav V. Verkhusha

**Affiliations:** 1 Department of Anatomy and Structural Biology and Gruss-Lipper Biophotonics Center, Albert Einstein College of Medicine, Bronx, NY 10461, USA; 2 Laboratory of Structural Dynamics, Stability and Folding of Proteins, Institute of Cytology, Russian Academy of Sciences, St. Petersburg 194064, Russia; 3 Department of Biophysics, Peter the Great St. Petersburg Polytechnic University, St. Petersburg 195251, Russia; 4 Department of Biochemistry and Developmental Biology, Faculty of Medicine, University of Helsinki, Helsinki 00290, Finland

## Abstract

Bioluminescence imaging became a widely used technique for noninvasive study of biological processes in small animals. Bioluminescent probes with emission in near-infrared (NIR) spectral region confer the advantage of having deep tissue penetration capacity. However, there are a very limited number of currently available luciferases that exhibit NIR bioluminescence. Here, we engineered two novel chimeric probes based on RLuc8 luciferase fused with iRFP670 and iRFP720 NIR fluorescent proteins. Due to an intramolecular bioluminescence resonance energy transfer (BRET) between RLuc8 and iRFPs, the chimeric luciferases exhibit NIR bioluminescence with maxima at 670 nm and 720 nm, respectively. The 50 nm spectral shift between emissions of the two iRFP chimeras enables combined multicolor bioluminescence imaging (BLI) and the respective multicolor fluorescence imaging (FLI) of the iRFPs. We show that for subcutaneously implanted cells, NIR bioluminescence provided a 10-fold increase in sensitivity compared to NIR FLI. In deep tissues, NIR BLI enabled detection of as low as 10^4^ cells. Both BLI and FLI allowed monitoring of tumor growth and metastasis from early to late stages. Multimodal imaging, which combines concurrent BLI and FLI, provides continuous spatiotemporal analysis of metastatic cells in animals, including their localization and quantification.

*In vivo* bioluminescence imaging (BLI) is a powerful and simple technique for studies of living animals and cells^1^,^2^,^3^. It is widely used for interrogating various ongoing biological processes such as tracking of luciferase-labeled cells, monitoring gene expression, assessing protein stability and function, and sensing small bioactive molecules. The mechanism of bioluminescence is based on substrate oxidation catalysis by luciferases to produce an excited-state species of a substrate that decay and emit photons of visible light. Thus, the bioluminescence mainly depends on the substrate concentration and the amount of a luciferase. The absence of excitatory light leads to lower background and hence higher sensitivity of bioluminescence-based detection, as opposed to fluorescence imaging (FLI). Conversely, BLI has several drawbacks e.g. a limited set of available colors and complicated spectral resolution of multiple bioluminescent probes in a single animal. Thereby, there is a room for optimization and improvement to fill a growing demand for sensitive multimodal imaging^4^.

One of the ways to expand the spectral diversity and sensitivity of BLI is designing fusions of luciferases and fluorescent proteins (FPs). The resulting chimeric constructs can have a significantly increased brightness due to bioluminescence resonance energy transfer (BRET)^5^,^6^. However, currently used constructs are limited by emission spectra of donor luciferases that are commonly selected for their development (e.g., *Renilla* luciferase (RLuc) and Firefly luciferase (FLuc)). For that reason, recently published constructs in that category cover only the spectral region from 474 nm of CNL^5^ to 635 nm of TurboRFP^7^,^8^. The superior probe for *in vivo* application therefore should not only merge the benefits of increased sensitivity of bioluminescence with the higher spatial and temporal resolution of fluorescence, but also have a near-infrared-shifted emission spectrum. The near-infrared (NIR) spectral range is preferable for deep-tissue and whole-body imaging due to both reduced light scattering and combined absorbance of hemoglobin, melanin and water^9^.

Here, we addressed several of the bioluminescence drawbacks and exploited both the use of modified substrate and the BRET mechanism in order to develop advanced NIR bioluminescent constructs. As a donor-acceptor pair for NIR luciferase we chose an enhanced version of *Renilla* luciferase, RLuc8^10^, and bacterial phytochrome-based NIR fluorescent proteins (iRFPs^11^). RLuc8 is a small (35.7 kDa), stable, bright and ATP-independent luciferase with a variety of available substrates, which makes it an advantageous donor of bioluminescence for *in vivo* imaging. In turn, the phytochrome-based FPs are well known as superior probes for NIR *in vivo* FLI^9^ with considerable potential for improvement of their performance characteristics^12^,^13^. A second absorption peak at around 380 nm, known as a Soret band, made iRFPs favorable acceptors of RLuc8 bioluminescence (Fig. 1a). Using iRFP670 and iRFP720 we engineered a pair of spectrally distinct NIR chimeric luciferases that we further tested in multicolor BLI and FLI in cells and in mice. Lastly, we proceeded to demonstrate that high sensitivity of bioluminescence combined with NIR fluorescence enables continuous and sustained analysis of cellular processes on different scales, from isolated cells to whole organs, using the same NIR chimeric probes.

## Results

### Design and development of NIR luciferases

**Figure 1:**
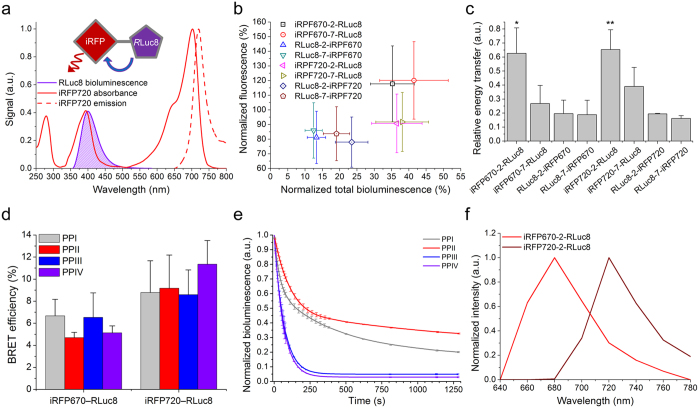
Engineering and characterization of two-color near-infrared luciferases. (**a**) Schematic of the domain structure of chimeric luciferase and bioluminescence resonance energy transfer between RLuc8 and iRFP720. (**b**) NIR fluorescence signal of live HeLa cells transiently transfected with the NIR chimeric luciferase constructs, detected by flow cytometry and normalized to brightness of the respective iRFP protein and plotted against total bioluminescence of HeLa cells lysates with PPII substrate normalized to brightness of RLuc8. (**c**) Relative BRET between RLuc8 and FPs for each construct obtained by dividing NIR bioluminescence signal at the optimal wavelength by total bioluminescence signal obtained with PPII substrate. (**d**) BRET efficiency for iRFP670—RLuc8 and iRFP720—RLuc8 measured with different substrates. (**e**) Bioluminescence reaction kinetics of different substrates catalyzed by iRFP720—RLuc8 at 25 °C *in vitro*. (**f**) Bioluminescence spectra of iRFP670—RLuc8 and iRFP720—RLuc8 measured using 20 nm bandpass filters. Error bars, s.d. (n = 3). *P < 0.05 (*t*-test) versus iRFP670-7-RLuc8, RLuc8-2-iRFP670 and RLuc8-7-iRFP670. **P < 0.05 (*t*-test) versus iRFP720-7-RLuc8, RLuc8-2-iRFP720 and RLuc8-7-iRFP720.

In the chimeric protein consisting of a luciferase and an iRFP protein the intramolecular BRET occurs via Soret band (Fig. 1a), resulting in NIR emission at iRFP spectral maximum. To find chimeric constructs with the highest NIR bioluminescence signal, we tested several fusions between RLuc8 and iRFP670 and iRFP720 proteins^11^ varying the order of both proteins and length of the linker between them. This allowed us to vary the distance between the active site of the luciferase and the chromophore of the fluorescent protein, thus changing effectiveness of the intramolecular energy transfer. We first compared brightness of fluorescence and total bioluminescence of the chimeric constructs normalized to the brightness of the respective iRFP protein and RLuc8, respectively (Fig. 1b). We then estimated relative BRET ratio for each construct by dividing NIR bioluminescence of the acceptor to bioluminescence of the donor in both cases obtained by narrow emission filters (Fig. 1c). Resulting data clearly show that iRFPs—RLuc8 chimeric constructs provided higher signal than RLuc8—iRFPs constructs. Additionally, the constructs with linkers consisting of two amino acid residues exhibited substantially higher BRET efficiency than the constructs with linkers consisting of seven amino acid residues. Therefore, for further experiments we chose iRFP670—RLuc8 and iRFP720—RLuc8 both having the shortest linkers.

To match the absorption spectrum of iRFPs’ Soret band in preliminary tests we used a PPII substrate with the most violet-shifted spectrum of bioluminescence with RLuc8 (~400 nm, Table 1). However, since both the high spectral overlap and brightness of the substrate are critical for BRET, we tested other available substrates for short wavelength light emission, which were 5 or 10 nm red-shifted, relative to the PPII substrate (Table 1). To compare coelenterazine-based substrates *in vitro* we measured BRET efficiency (Fig. 1d), bioluminescence kinetics (Fig. 1e) and stability in PBS (Table 1). We found no significant difference between BRET efficiencies of the tested substrates. However, the average BRET efficiency of iRFP670—RLuc8 with all the tested substrates was lower than that of iRFP720—RLuc8, which is the result of the higher spectral overlap between bioluminescence spectrum and Soret band of iRFP720. Bioluminescence half-lives of PPI and PPII was substantially longer (153 s and 217 s, respectively) as compared to that of PPIII and PPIV (~48 s for both). Summarizing the data, for further experiments we chose PPI as the stable and bright substrate for *in vitro* applications.

We next performed spectral characterization of iRFP670—RLuc8 and iRFP720—RLuc8 *in vitro* using the set of 20 nm band-pass emission filters (Fig. 1f). We verified that NIR bioluminescence spectra shape and peak positions are similar to the fluorescence emission spectra of the respective iRFPs^11^. Hence the resulted shift between the donor and acceptor emissions was up to 315 nm (RLuc8 emission at 405 nm; iRFP720 emission at 720 nm).

### Multicolor and multimodal imaging in cells

**Figure 2:**
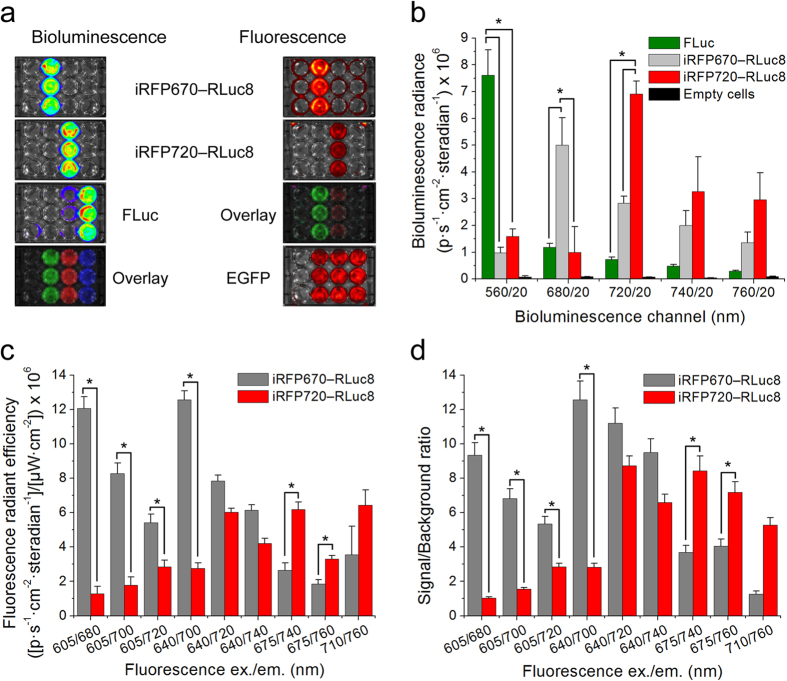
Cell-based multicolor and multimodal imaging of HeLa cells. (**a**) Representative bioluminescence and fluorescence images of 12-well plates with HeLa cells expressing iRFP670—RLuc8, iRFP720—RLuc8 or FLuc and co-expressing EGFP after unmixing of signals. (**b**) Quantification of the bioluminescence signals for the images in (**a**). (**c**) Quantification of the fluorescence signals with subtracted background for the images in (**a**). (**d**) Quantification of the fluorescence signal-to-background ratios for the images in (**a**). Error bars, s.d. (n = 3). *P < 0.05 (*t*-test).

We next demonstrated multicolor BLI and FLI with high contrast in HeLa cells. We imaged 12-well plate using IVIS Spectrum imager equipped with cooled charge-coupled device (CCD) camera capable of measuring both fluorescence and luminescence in a wide spectral range (Fig. 2). For this we transiently transfected HeLa cells with two plasmids, one encoding NIR chimeric luciferase or FLuc and another encoding EGFP as a control. Applying a standard linear spectral unmixing procedure we imaged three bioluminescent (iRFP670—RLuc8, iRFP720—RLuc8 and FLuc) and three fluorescent colors (iRFP670—RLuc8, iRFP720—RLuc8 and EGFP) (Fig. 2a). To compare multicolor performance we calculated imaging contrast of BLI and FLI in individual channels defined as the ratio between the signals of two constructs in the same channel. In this way, the imaging contrast numbers represent the ability to unmix the signals of two constructs using a specific setup. The BLI using chimeric luciferases had contrast of 5.0-fold and 2.5-fold for the 680/20 nm and 720/20 nm emission filters, respectively (Fig. 2b), resulting in 12.5-fold contrast between two bioluminescence channels. Applying a combination of the excitation and emission filters for FLI we achieved contrast of 9.5-fold and 2.3-fold for the 605/680 nm and 675/740 nm excitation/emission filters combinations, resulting in 21.9-fold contrast between the two fluorescence channels (Fig. 2c). Thus, the combined contrast of both BLI and FLI of NIR chimeric luciferases can reach ~274-fold. Furthermore, both NIR chimeric proteins had high signal-to-autofluorescence ratios (Fig. 2d).

### Minimal amount of cells detected by BLI and FLI

**Figure 3:**
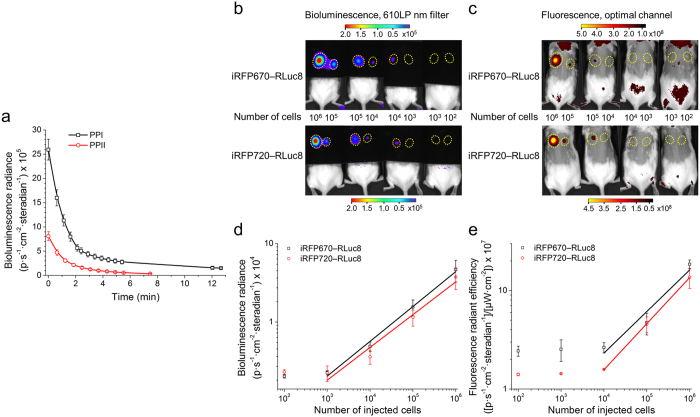
*In vivo* kinetics and comparative multimodal BLI and FLI of subcutaneously implanted cells. (**a**) Bioluminescence reaction kinetics of PPI and PPII substrates catalyzed by 10^6^ subcutaneously implanted MTLn3 cells stably expressing iRFP720—RLuc8. (**b**) BLI and (**c**) FLI of MTLn3 cells stably expressing iRFP670—RLuc8 or iRFP720—RLuc8 subcutaneously implanted in CFW mice (dorsal view). The color bars indicate the total bioluminescence radiance (photons s^−1^ cm^−2^ steradian^−1^) or the total fluorescence radiant efficiency (photons s^−1^ cm^−2^ steradian^−1^ per μW cm^−2^). The yellow circles represent regions of interest (ROI) used for data quantification. (**d**) Values of bioluminescence signal for the images in (B). (**e**) Values of fluorescence signal for the images in (**c**). The black and red lines represent linear approximation of dependence of logarithm of the signal strength on logarithm of the number of cells. Error bars, s.d. (n = 6).

To determine sensitivity of NIR chimeric luciferases and compare their abilities in BLI and FLI we subcutaneously implanted or injected via tail vein the rat mammary adenocarcinoma MTLn3 cells stably expressing either iRFP670—RLuc8 or iRFP720—RLuc8 into CFW mice. However, before proceeding to this set of experiments we sought to choose the finest substrate for *in vivo* use from the two best substrates according to *in vitro* tests (PPI and PPII). For this we measured both kinetics and brightness of 10^6^ subcutaneously implanted iRFP720—RLuc8 expressing MTLn3 cells for each substrate (Fig. 3a). Both substrates had similar kinetics, yet *in vivo* PPI was 3.2-fold brighter, making it preferable for *in vivo* experiments.

**Figure 4:**
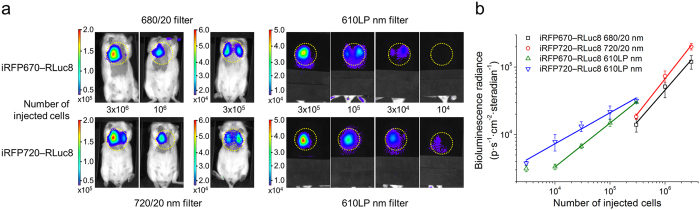
Deep-tissue BLI with NIR chimeric luciferases. (**a**) Imaging of MTLn3 cells stably expressing iRFP670—RLuc8 or iRFP720—RLuc8 injected in CFW mice via tail vein (ventral view). The color bars indicate the total bioluminescence radiance (photons s^−1^ cm^−2^ steradian^−1^) or the total fluorescence radiant efficiency (photons s^−1^ cm^−2^ steradian^−1^ per μW cm^−2^). The yellow circles represent regions of interest (ROI) used for data quantification. (**b**) Values of bioluminescence signal for the images in (A). The blue, green, black and red lines represent linear approximation of dependence of logarithm of the signal strength on logarithm of the number of cells. Error bars, s.d. (n = 5).

At first, to estimate the minimal amount of cancer cells detected subcutaneously we implanted from 10^2^ to 10^6^ MTLn3 cells and acquired bioluminescent and fluorescent images 2 h after implantation (Fig. 3b,c). According to the data, the limit of detection by BLI, using PPI as a substrate, was between 10^3^ and 10^4^ cells stably expressing either iRFP670—RLuc8 or iRFP720—RLuc8 (Fig. 3d), which was 10-fold lower than the number of the same cells detected using FLI (Fig. 3e). Secondly, to determine the minimal amount of cancer cells detected deeply in tissues we injected via tail vein from 3 × 10^3^ to 3 × 10^6^ MTLn3 cells and acquired images 1 h after injection. Using narrow 20 nm band-pass emission filters and spectral unmixing we could detect as few as 3 × 10^4^ cells located in the lungs (Fig. 4a). However, imaging of the total light output (open filter) allows detector to collect more photons and routinely used to lower the detection limit of BLI. To demonstrate that the sensitivity of NIR chimeric luciferases relies only on NIR spectral component, we excluded the light of shorter wavelengths by covering mice with a large-area 610 LP nm filter. Thus, the NIR bioluminescence of iRFP670—RLuc8 and iRFP720—RLuc8 allowed detection of 3 × 10^4^ and 10^4^ cells located in the lungs of living mice, respectively (Fig. 4b).

**Figure 5:**
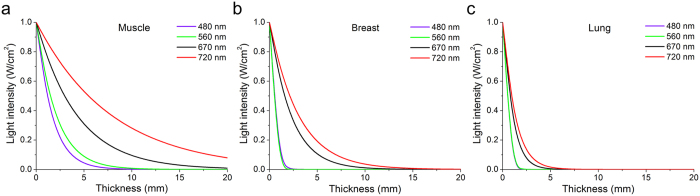
Comparison of the attenuation of the visible and NIR light in different mammalian tissues. Dependences of the light intensities at 480 nm, 560 nm, 670 nm and 720 nm wavelengths on the thickness of (**a**) muscle, (**b**) breast and (**c**) lung tissues were calculated as described in the Methods.

To explain the variation between the sensitivity of NIR chimeric luciferases we sought to compare optical properties of mammalian tissues in different parts of the spectrum. First, based on the published experimental data^14^,^15^,^16^ we calculated effective attenuation coefficients for two NIR wavelengths (670 and 720 nm) and for two visible spectral range wavelengths (482 and 560 nm). We chose the latter according to the emission maxima of luciferases most commonly used in BLI: RLuc8 with coelenterazine substrate and FLuc with D-luciferin substrate. Secondly, we plotted the dependence of light intensity at different wavelengths on the thickness of three types of mammalian tissue, such as muscle, breast and lung (see Methods). Lastly, we compared half-value thickness (HVT) of each tissue for different wavelengths. In muscle tissue, as the most transparent among the studied, HVT of 720 nm light was 1.9-fold, 3.9-fold and 5.0-fold greater than HVT of 670 nm, 560 nm and 482 nm light, respectively (Fig. 5a). In breast tissue containing a large amount of lipids difference of HVT between 720 nm and 670 nm light was only 1.4-fold. At the same time HVT of 720 nm light in breast was 12.9-fold and 8.6-fold greater than HVT of 560 nm and 480 nm light, respectively (Fig. 5b). The lungs, as the most complex and heterogeneous tissue, had the highest attenuation coefficients. HVT of 720 nm light was 1.3-fold, 4.3-fold and 4.8-fold greater than HVT of 670 nm, 560 nm and 482 nm light, respectively (Fig. 5c). These estimations provide an explanation of the difference in the sensitivity of NIR chimeric luciferases. In addition, the data suggests that deep-tissue BLI using visible spectral range bioluminescence should be at least 4-fold brighter than NIR bioluminescence to provide the same sensitivity.

### Imaging of tumors expressing NIR luciferases in mice

**Figure 6:**
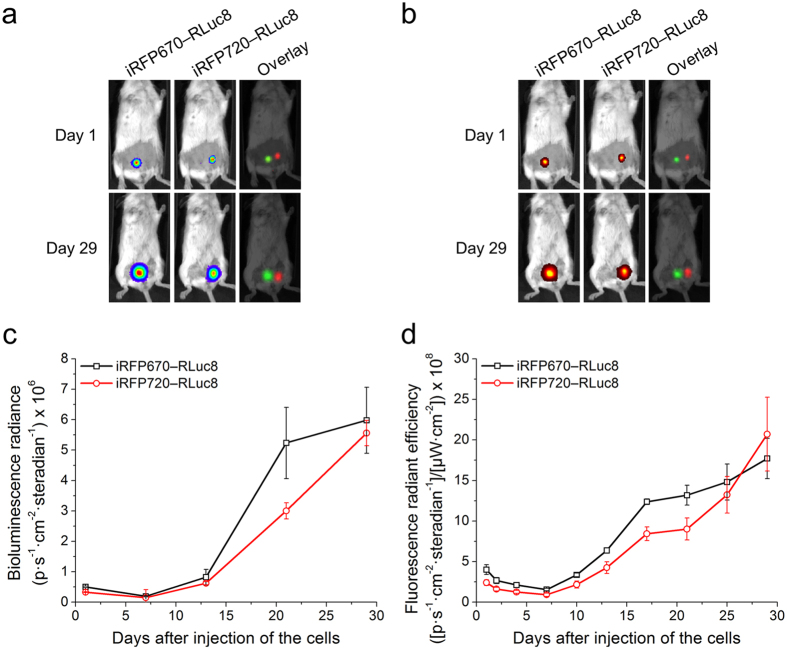
Growth of xenograft tumors expressing iRFP670—RLuc or iRFP720—RLuc in mice. (**a**) BLI and (**b**) FLI of 2.5 × 10^6^ MTLn3 cells orthotopically implanted into the mammary fat pads of mice with images taken on the 1st and 29th day of tumor growth (dorsal view). (**c**) Values of bioluminescence signal for the images in (**a**). (**d**) Values of fluorescence signal for the images in (**b**). Error bars, s.d. (iRFP670—RLuc8 tumors n = 5, iRFP720—RLuc8 tumors n = 6).

To grow primary xenograft tumors we used mice with severe combined immunodeficiency (SCID). MTLn3 cells stably expressing either iRFP670—RLuc8 or iRFP720—RLuc8 were orthotopically implanted into the mammary fat pads on the left and right sides of the animals, respectively. We monitored tumor growth for 29 days by BLI and FLI (Fig. 6). Both imaging techniques allowed us to spectrally unmix the signals of cells expressing iRFP670—RLuc8 and iRFP720—RLuc8 from early to late stages and showed similar kinetics of tumor growth (Fig. 6a,b). Interestingly, we observed that the iRFP670—RLuc8 expressing tumors grew faster than the iRFP720—RLuc8 ones despite an equal amount of initially implanted cells (Fig. 6c,d). For instance, both BLI and FLI detected 1.2-fold faster growth of iRFP670—RLuc8 tumors during the period between 13^th^ and 21^st^ days after cell implantation. However, the difference in total bioluminescence and fluorescence signals between the tumors significantly decreased to 29^th^ day of measurement.

### Visualization of metastasis *in vivo* and *ex vivo*

**Figure 7:**
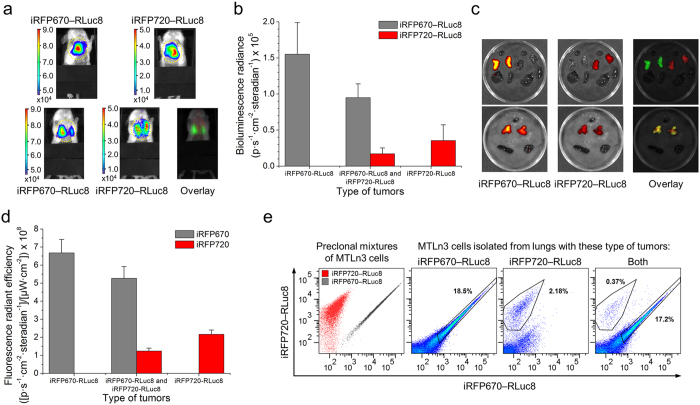
iRFP670—RLuc and iRFP720—RLuc tumor metastasis in mice. (**a**) BLI of metastasis in the lungs of mice with single (top row) or two different (bottom row) tumors (ventral view). The color bars indicate the total bioluminescence radiance (photons s^−1^ cm^−2^ steradian^−1^). The yellow circles represent regions of interest (ROI) used for data quantification. (**b**) Values of bioluminescence signals for the images in (**a**). (**c**) *Ex vivo* FLI of the internal organs of mice with one (top row) or two different (bottom row) tumors. (**d**) Values of fluorescence signal for the images in (**c**). (**e**) Representative FACS plots showing separation of MTLn3 preclonal mixtures stably expressing iRFP670—RLuc8 or iRFP720—RLuc8 and MTLn3 cells isolated from the lungs of mice. The gates were selected according to the brightness of MTLn3 preclonal mixtures. The numbers represent the relative average amount of cells in gates. Error bars, s.d. (n = 3).

Our objective was to demonstrate deep-tissue imaging that requires high brightness, low absorption and scattering of light signals in tissues. Because of a limited dynamic range associated with CCD cameras, imaging of deep-seated metastasizing cancer cells was only possible when the primary tumors were covered^17^. Thus by covering tumors in our experiments, we were able to detect lung metastasis using BLI (Fig. 7a). In the mice with both tumors we could detect not only the total signal coming from both iRFP670—RLuc8 and iRFP720—RLuc8 expressing cells, but successfully spectrally unmix two NIR signals. To determine the contribution of bioluminescence signals of each tumor type in mice containing both MTLn3 expressing iRFP670—RLuc8 and MTLn3 expressing iRFP720—RLuc8 tumors we imaged mice with only single type of tumor as well. To quantify the amount of migrated tumor cells in the lungs we used calibration curves obtained from previous experiment (Fig. 4b) with the same cells. We calculated that the total bioluminescence brightness of the lungs region (Fig. 7b) corresponded to the emission of 3.7 × 10^6^ of iRFP670—RLuc8 positive cells (±9.52 × 10^5^) and 5.42 × 10^5^ of iRFP720—RLuc8 positive cells (±3.34 × 10^5^) in mice with single type of tumor. The amount of iRFP670—RLuc8 and iRFP720—RLuc8 cells in mice with both types of tumors was estimated to be 2.19 × 10^6^ (±3.91 × 10^5^) and 2.7 × 10^5^ (±1.27 × 10^5^), respectively.

To evaluate metastatic cell distribution and to test *ex vivo* potential of NIR chimeric luciferases we dissected the mice. The ability of chimeric probes to be used in multimodal imaging enabled assess to *ex vivo* cell distribution using FLI and fluorescence-activated cell sorting (FACS). We first isolated internal organs of the mice including heart, lungs, liver and spleen (Fig. 7c). Then we estimated the average intensity of NIR fluorescence signals for each chimeric construct (Fig. 7d), using heart, an organ with high blood content and rarely contaminated with metastasis, as a control for autofluorescence. By means of FLI we detected difference in the signal intensities of iRFP670—RLuc8 and iRFP720—RLuc8 similar to the data obtained on intact mice by BLI (Fig. 7b). Next, to examine metastasis at a more precise level we mechanically disaggregated lung tissues. The resulted suspensions of lung cells were analyzed using FACS. The resulting dot plots clearly show (Fig. 7e), that all the extracted samples contained metastatic cells. The brightness of these cells matched that of the original injected MTLn3 cells, suggesting that the NIR chimeras are non-toxic to cells and retain high expression levels even after 4 weeks in the mice.

## Discussion

The expansion of spectrally distinct bioluminescent constructs with enhanced light-emitting properties is an extensive area of research. Multiple approaches have been applied in order to shift the bioluminescence spectrum of luciferases. For example, the mutagenesis of RLuc and FLuc, with natural peak emissions at 482 nm and 560 nm, resulted in shifting of bioluminescence to 547 nm^18^ and 677 nm (Red-FLuc, PerkinElmer), respectively. The chemical modification of natural substrates allowed shifting of FLuc mutant peak emission to 677 nm^19^ or even 706 nm^20^; however, these new substrate require a complex process of chemical synthesis and are not commercially available. The conjugation of luciferases with inorganic compounds such as AF750 dye^21^ and quantum dots^22^ resulted in a greater shift of bioluminescence spectra via BRET mechanism (peak emission at 783 nm and 800 nm, respectively). Yet the conjugation of inorganic compounds with luciferases significantly limits their range of *in vivo* applications because of their potential toxicity and the inability to produce these probes in living cells.

Thus, repeating the path of fluorescent proteins over the past decade, development of NIR-shifted sources of bioluminescence for *in vivo* imaging becomes important. Using novel coelenterazine-based substrates for violet light emission of RLuc8 and optimizing iRFPs—RLuc8 fusions, we have developed two-component NIR chimeric luciferases for multicolor multimodal BLI and FLI techniques. Due to high contrast and narrow emission peaks of the engineered NIR luciferases, they can be multiplexed with other fluorescent proteins and luciferases, such as FLuc, NanoLuc^6^,^23^, Lumifluors^6^ and Nano-Lanterns^5^, greatly expanding the palette of *in vivo* colors.

To assess the characteristics of our new multimodal tools, we used a well-established mammary adenocarcinoma tumor model already applied for drug development, metastasis studies and *in vivo* optical probe characterization^24^,^25^,^26^,^27^. We found that the NIR emission spectra of the chimeras due to efficient BRET from RLuc8 to iRFPs enable quantitative *in vivo* skin-deep BLI and FLI and deep-tissue BLI of tumor cells. Despite the lack of quantitative deep-tissue BLI data, with which we can compare our results, BLI with NIR chimeric luciferases reaches the level of detection of such sensitive optical techniques as fluorescence lifetime imaging (FLIM)^28^ and reversibly switchable photoacoustic computed tomography (RS-PACT)^29^. For example, FLIM could reach sensitivity of 1.4 × 10^3^ subcutaneously injected cells and 5.0 × 10^4^ cells dispersed in nude mouse lungs. Yet in contrast to FLIM and RS-PACT, performing BLI with our chimeras requires standard commercial equipment and no additional computation, making it more affordable and suitable for a wider range of applications.

NIR chimeric luciferases provide the necessary level of sensitivity for non-invasive detection of tumor growth and metastasis in living mice. Moreover, the fluorescent component of NIR chimeric luciferases permits isolation and analysis of dispersed cells on a single-cell scale by FACS or microscopy. This additional capacity could be useful for determination of tumor cell phenotypes and studying cancer evolution^30^. Currently, iRFP720—RLuc8 is the most NIR-shifted genetically encoded bioluminescent probe. We anticipate that the NIR bioluminescent chimeras will become the probes of choice for a variety of *in vivo* studies.

## Methods

### Design of NIR chimeric luciferases

PCR-amplified *AgeI-KpnI* fragments encoding iRFP670 or iRFP720 and *KpnI-NotI* fragment encoding RLuc8 (Addgene #51970)^31^ were swapped with a gene encoding EGFP in a pEGFP-N1 vector (Clontech), resulting in piRFP670—RLuc8-N1 and piRFP720—RLuc8-N1 plasmids. For cloning of fragments in reverse order a PCR-amplified *AgeI-KpnI* fragment encoding RLuc8 and *KpnI-NotI* fragments encoding iRFP670 or iRFP720 were used. Primers with a *KpnI* restriction site at the end encoded the linker between proteins. The linker consisted of two amino acid residues (-GT-), derived from *KpnI* restriction site or 7 amino acid residues (-GGGGSGT-). A PCR-amplified *AgeI-NotI* fragments encoding RLuc8 or FLuc were swapped with a gene encoding EGFP in a pEGFP-N1 vector.

### Cell culture

HeLa cell lines were grown in DMEM containing 10% FBS, 0.5% penicillin-streptomycin and 2 mM glutamine (Life Technologies/Invitrogen). MTLn3 rat adenocarcinoma cells were cultured in αMEM medium (Life Technologies/Invitrogen) supplied with 5% FBS, 0.5% penicillin-streptomycin and 2 mM glutamine (Life Technologies/Invitrogen). Plasmid transfections were performed using an Effectene reagent (Qiagen) according to the manufacturer’s protocol. Stably expressing cells were selected with 700 μg/ml G418 antibiotic. Sorting of positive cells was performed using a MoFlo XDP sorter (Beckman Coulter) equipped with a 633 nm HeNe and a 676 nm Kr lasers and a 700 LP nm emission filter.

### Protein characterization *in vitro*

To measure total bioluminescence, relative BRET, BRET efficiency, bioluminescence kinetics and spectra of NIR chimeric constructs we obtained HeLa cells lysates 48 h after transfection with NIR chimeric luciferases and co-transfected with pEGFP-N1 to normalize for transfection efficiency and average amount of protein in the sample. To extract protein of interest form HeLa cells freeze-thaw procedure was used. To measure bioluminescence, 20 μl of cell lysate were mixed with 20 μl of 50 μM substrate solution in PBS (NanoLight Technologies) in a well of a 96-well plate. All measurements of bioluminescence and fluorescence were made using the IVIS Spectrum and the Living Image v. 4.3.1 software (PerkinElmer/Caliper). The total bioluminescence was measured using open filter. To measure relative BRET for each NIR chimeric luciferase the bioluminescence intensity in the optimal NIR emission channel (680/20 nm for chimeric constructs based on iRFP670 and 720/20 nm for chimeric constructs based on iRFP720) was divided by the bioluminescence intensity in the most blue-shifted available channel 500/20 nm corresponding to bioluminescence of donor RLuc8. The BRET efficiency was quantified according to recommendations described here^7^ with minor modifications: BRET efficiency = 100 × [Fusion Reporter Bioluminescent emission (610 LP nm filter (Chroma))/Bioluminescent emission (open filter)] – [Donor Only Bioluminescent emission (610LP nm filter)/Donor Only Bioluminescent emission (open filter)]. All quantitative measurements of bioluminescence and fluorescence signals were performed using the Living Image v. 4.3.1 software (PerkinElmer/Caliper).

### Flow cytometry

For flow cytometry analysis of effective brightness in HeLa cells, the piRFP—RLuc8-N1 plasmids encoding NIR chimeric luciferases and piRFP-N1 plasmids encoding iRFP670 or iRFP720 were co-transfected with pEGFP-N1 to normalize for transfection efficiency. Fluorescence intensity of cells was measured 48 h after transfection using a BD LSRII flow cytometer (BD Biosciences) equipped with 488 nm Ar and 640 nm solid-state lasers and using 530/30 nm, 660/20 nm and 730/30 nm emission filters.

For cell fluorescence quantification, a mean fluorescence intensity of the non-negative population in the near-infrared channel was divided by a mean fluorescence intensity of the same population in the green channel, thus normalizing the near-infrared signal to the transfection efficiency. All FACS calculations were performed using the FlowJo software (Tree Star).

### Cell imaging

For multicolor and multimodal imaging of HeLa cell cultures, the piRFP—RLuc8-N1 plasmids encoding NIR chimeric luciferases and pFLuc-N1 were cotransfected with pEGFP-N1 to normalize for transfection efficiency. Bioluminescence and fluorescence imaging was performed with IVIS Spectrum (PerkinElmer/Caliper). To compare bioluminescence, average radiance intensity of transfected 12-well plates (with their lids removed) was determined using different emission filters. To compare fluorescence, radiant efficiency was determined with different excitation and emission filter sets. For bioluminescence measurements 200 μl of 50 μM PPI substrate solution in PBS (NanoLight Technologies) or 200 μl of 30 μg/ml D-luciferin substrate were added to cells per well. Both bioluminescence and fluorescence data were corrected for background and normalized for transfection efficiency. This was obtained by subtracting the average signal background (measured from the empty vector-containing wells) from the bioluminescence or fluorescence flux of each luminescent protein-containing well, divided by its EGFP fluorescence.

### Calculation of the optical properties of tissue

To determine the effective attenuation coefficient (*μ*_*eff*_) we used approach described previously^32^. Using literature data, we found the values of reduced scattering coefficient (*μ′*_*s*_) and absorption coefficient (*μ′*_*a*_) for three types of tissues. Then using this equation


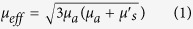
we obtained the following values of μ_*eff*_ (mm^−1^): muscle (*μ*_*eff*_^480^ = 0.63, *μ*_*eff*_^560^ = 0.49, *μ*_*eff*_^670^ = 0.24, *μ*_*eff*_^720^ = 0.13)^14^, breast (*μ*_*eff*_^480^ = 2.86, *μ*_*eff*_^560^ = 4.31, *μ*_*eff*_^670^ = 0.45, *μ*_*eff*_^720^ = 0.33)^15^, lung (*μ*_*eff*_^480^ = 4.11, *μ*_*eff*_^560^ = 3.66, *μ*_*eff*_^670^ = 1.10, *μ*_*eff*_^720^ = 0.85)^16^. To assess the degree of attenuation depending on the thickness of the tissue we used Beer–Lambert–Bouguer law equation


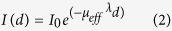
where *I*_*0*_ is the initial light intensity (W/cm^2^), *μ*_*eff*_^*λ*^ is the effective attenuation coefficient of tissue at wavelength λ (mm^−1^), and *d* is the path length of light through the sample (mm).

### *In vivo* whole body imaging

All animal experiments were performed in AAALAC approved facility in accordance with current guidelines and regulations using protocols approved by the Albert Einstein College of Medicine Animal Usage Committee. Mice were housed in a vivarium on a 12 h light-dark cycle at five mice per cage. Only female mice were used in this study.

MTLn3 cells stably expressing either iRFP670—RLuc8 or iRFP720—RLuc8 were dissociated via trypsin digestion, and suspended in RPMI media. Desired amounts of MTLn3 cells were either subcutaneously implanted or injected via tail vein in CFW mice (4–6 weeks old Charles River), or injected into the mammary gland of SCID/NCr mice (4–6 weeks old, Taconic). The imaging started after 1 h for quantitative experiments or after a day for monitoring of tumor grows using the IVIS Spectrum. The fur was removed using a depilatory cream. Before imaging mice were anesthetized (2% isoflurane oxygen), and 0.7 mg/kg of mice body weight of substrate in PBS was intravenously administered. Immediately after injection mice were imaged in four bioluminescence channels, with up to 2 min acquisition time at each 20 nm bandpass channel or up to 5 min in open filter channel. For FLI all mice were imaged in 18 filter channels. Regions of interest were drawn over the mice mammary gland area or lungs area for each image. The signal from control mice injected only with the substrate was subtracted from the total signal. All quantitative measurements of bioluminescence and fluorescence signals as well as their linear spectral unmixing were performed using the Living Image v. 4.3.1 software (PerkinElmer/Caliper).

For bioluminescence and fluorescence time-course measurement, mice with a single tumor or two different tumors were imaged over 4 weeks after cell injection. Background subtracted images of average radiance intensities or total radiant efficiencies of the same region over tumors were generated. Filter channels used for calculation of tumor growth curves were the following: 640/30 nm exciter and 680/20 nm emitter for iRFP670—RLuc8, and 675/30 nm exciter and 720/20 nm emitter for iRFP720—RLuc8.

### *Ex vivo* lungs imaging and flow cytometry

The internal organs of mice were excised postmortem and FLI was performed using the IVIS Spectrum as described above. For flow cytometry analysis of metastatic cells in the lungs they were mechanically disaggregated. The lungs were chopped into 2–3 mm diameter pieces and mixed with PBS containing 2% of BSA. The resulted suspension was filtered first through 70 μm and after through 40 μm nylon cell strainers. To lyse red blood cells the suspension of filtered cells was pelleted and resuspended in PBS solution supplemented with 1–6% NaCl. After 30 s incubation the cells were washed with PBS containing 2% of BSA and analyzed using the BD LSRII flow cytometer (BD Biosciences) as described above.

### Statistical analysis

Data were statistically analyzed with a two-sided Student’s t-test and presented as means ± s.d. The P-values < 0.05 were considered statistically significant.

## Additional Information

**How to cite this article**: Rumyantsev, K. A. *et al*. Near-infrared bioluminescent proteins for two-color multimodal imaging. *Sci. Rep.* **6**, 36588; doi: 10.1038/srep36588 (2016).

**Publisher’s note:** Springer Nature remains neutral with regard to jurisdictional claims in published maps and institutional affiliations.

## Figures and Tables

**Table 1:** Properties of the substrates resulting in RLuc8 bioluminescence in a violet wavelength range.

Name of the substrate	Prolume Purple (PPI)	Prolume Purple II (PPII)	Prolume Purple III (PPIII)	Prolume Purple IV (PPIV)
Alternative name	Methoxy-eCoelenterazine (me-eCTZ)	Methoxy-eCoelenterazine-Methoxy (Me-CTZ-Me)	Methoxy-eCoelenterazine-F (Me-eCTZ-F)	Methoxy-Coelenterazine-Iodine (Me-CTZ-I)
Molecular formula	C_29_H_25_N_3_O_3_	C_28_H_25_N_3_O_3_	C_29_H_24_FN_3_O_2_	C_27_H_22_IN_3_O_2_
Molecular weight	463.5 Da	451.5 Da	465.5 Da	547.4 Da
Emission maximum with RLuc8	405 nm	400 nm	410 nm	410 nm
Stability in phosphate buffered saline at 25 °C	relatively stable (>20 min)	relatively stable (>15 min)	unstable (<10 min)	unstable (<10 min)
Brightness relative to Coelenterazine 400a substrate (also called as DeepBlueC)	13-fold brighter	5-fold brighter	10-fold brighter	no data
